# Adhesion of the Soft Palate and Uvula to the Posterior Wall of the Pharynx—Operation—Cure

**Published:** 1873-05

**Authors:** A. B. Cooke


### ARTICLE V.

### *Adhesion of the Soft Palate and Uvula to the Posterior Wall of the Pharynx—Operation—Cure.*

BY A. B. COOK, M. D.

The subject of the following case, F—— J——, woman of color, low, heavy set, with short and thick neck, 27 years of age, married, the mother of two children, the youngest now 12 years old and healthy, some years ago contracted syphilis, at what time cannot now be ascertained. Previous to the spring of 1870 she was a stout, healthy woman. About that time her throat became ulcerated, which was the first evidence of secondary syphilis; the ulceration involved, so far as I can learn, the tonsils, soft palate, and upper portion of the pharynx, accompanied with tumefaction and elongation of the uvula. She stated that from the spring of 1870 until last June she was almost constantly under medical treatment, which embraced a great variety of internal medication, with the local application of caustics, gargles, probanging, and inhalations, despite all which the disease persisted and produced the following local structural changes, lesions, and symptoms which presented when I was first called to see her in May, 1872.

The soft palate was firmly adherent to the pharynx, excepting a diminutive opening in the left side of the median line, scarcely large enough to admit the passage of an ordinary sized probe.

The uvula was broad, flat, enlarged, and elongated, the apex reaching a point corresponding to the inlet of the larynx; it was inclined a little to the right of the median line, and was completely attached to the posterior wall of the pharynx, the right posterior palatine arch, and the posterior part of the right tonsil. She complained of great soreness on the floor of the posterior nares, saying it felt like a raw place, due, no doubt, to a superficial ulcer. The pharynx and tonsils presented several superficial ulcers, which readily yielded to the local application of silver and mopping with a solution of chlorate of potash.

The adhesions first commenced to form in January, 1871, at which time she had daily hemorrhages for five weeks, the blood passing from the nose during the night, and from the bowels during the day, causing spanæmia and general debility. Instruments were frequently used by her physicians to tear up and prevent the adhesions, but to no useful purpose. For three months previous to the time I first saw her she had been unable to swallow any solid food; her diet was limited to toast water, tea, and very thin broths.

On June 10, 1872, assisted by Ed. T. Irwin, I dissected the uvula from the posterior wall of the pharynx. The tongue was depressed with a spatula by the assistant. I first made an incision with a long, straight-pointed tenotome, severing the right border of the uvula from its attachments to the right posterior palatine arch and right tonsil. I then dissected the uvula, from apex to base, with the angular-bladed knives used in staphyloraphy, from its attachment to the posterior wall of the pharynx. The hemorrhage, which was free, was easily arrested by the sponge mop saturated with water. In dissecting behind the base of the uvula, the small orifice in the soft palate was enlarged to admit the passage of a No. 10 flexible sound into the posterior nares.

I deemed it prudent to desist from further operative proceeding at that time, lest the violence done to the parts might result in sloughting, ulceration and destruction of the uvula and soft palate. I then directed the patient to mop several times daily with a weak solution of alum, taking care to pass the mop well up behind the uvula, to prevent any readhesion, and to inject the posterior nares with the same wash, using for this purpose a syringe having an angular point. The great anxiety of the patient to be cured induced her to faithfully carry out all directions.

In about three weeks the wound had healed, and the uvula, at first rather unsightly, had contracted and retracted to about the average size, and looked quite respectable.

The constitutional treatment at this time consisted of tr. ferri mur., min. 20 ; tr. cinchona comp., ʒ ij, in sweetened water, every four hours ; and hydrate chloral, grs. 15, in solution, at night, to produce sleep, which for a long time had been disturbed.

During the summer and fall she had repeated attacks of superficial ulceration of the pharynx and tonsils, which readily healed after a few applications of argenti nitras, or mopping with tr. ferri mur., diluted. The internal administration of iron was, every few weeks, alternated with the fluid ext. stillingiæ, ʒ ss ; potassi iod., grs. 4 ; hydrarg. biniodidi, gr. 1-20, in sweetened water, three times a day.

Under this treatment all disposition to ulceration of the throat, osseous pains and other general symptoms disappeared.

On the 10th day of November, assisted by J. A. Stratton, I detached the soft palate from the posterior pharyngeal wall. To reach this adhesion, and facilitate and simplify the operation, I had made, by F. S. Siegel, of this city, a special knife. The handle, of wood, is six inches long ; the shank, two inches ; the blade, one-half inch long, lancet shape, double edged, and set at a right angle with the shank, the edges being transverse to the long diameter of the shank. This knife I found admirably adapted for the purpose ; the blade being passed behind the uvula, the apex was forced through the soft palate, then the knife was carried transversely right and left, and the operation was completed in less time than is required to describe it. It was not necessary to use the tongue depressor, as the handle of the knife served for this purpose much better. The hemorrhage was slight and easily arrested. The next object of importance was to heal the wound, prevent any re-adhesion, and make the operation a success. I first bent the handle of a common tablespoon, passed it up behind the soft palate and determined the dimensions of the opening and extent of the passage. I next hammered out a piece of lead, from which I cut a plate one and a-half inches long and from three-fourths to seven-eighths of an inch in width, the lower end oval, the upper end notched in the centre, with a perforation in each cornua, the plate being slightly curved to rest on the posterior surface of the soft palate and uvula. A piece of hard corded surgeon's silk, about two feet in length, was tied in each opening through the corner of the cornua of the lead plate. I now passed a short piece of silk through the eye of a Belloc's cannula, making a short loop; then passed the cannula first through the left inferior meatis down behind the soft palate into the mouth, and attached one thread of the plate to the loop in the cannula and then withdrew the cannula as in plugging the posterior nares. The same was done in the right inferior meatus. I next grasped the lower border of the leaden plate with a pair of long forceps, carried it behind the uvula, at the same time drawing the silk threads through the nares with the left hand, when the plate was placed without difficulty in situ between the pharynx behind and the soft palate and uvula in front; the silk strings were passed above the ears and tied behind the head, drawn sufficiently tight to suspend the leaden plate so that the lower margin would correspond to the apex of the uvula. The patient was ordered to syringe the parts several times a day with a weak solution of alum. In a few days the parts were entirely healed, but in order to guard against contractions or re-adhesions I allowed the patient to wear the plate for six weeks, which she did with very little inconvenience, its presence not interfering with either deglutition or perspiration.

I cannot too strongly recommend the lead plate dressing in this and kindred operations; it is worn with ease and comfort, does not obscure the view of the parts at any time, facilitates thorough cleansing and the local application of remedies, and is an effectual barrier to any new adhesions.

This operation, or rather, the conditions requiring the operation, are of unfrequent occurrence. Many of our prominent authors on surgery do not refer to it in any way.

Dr. J. Solis Cohen, on "Diseases of the Throat"—for whose admirable work I am indebted to the publishers, William Wood & Co., 27 Great Jones street, New York—only mentions three cases: one described by Dr. Wm. Turner, 1860; one by Rudtroffer, in 1805, and one by Otto in 1813. Pieces of lint passed between the separated parts is the only dressing I find recommended to prevent re-adhesion, which, to say the least of it, must be a source of great annoyance and inconvenience to the patient.

Since the completion of the operation my patient has been able to swallow solids without difficulty. She complains of a want of action of the deglutory muscles on the right side of the pharynx, which is due to some contraction, being the sequel of ulceration previous to the time I first saw her. She has had no throat trouble during the winter. —*Med. and Surg. Reporter.*

---

